# Coadministration of Anti-Viral Monoclonal Antibodies With Routine Pediatric Vaccines and Implications for Nirsevimab Use: A White Paper

**DOI:** 10.3389/fimmu.2021.708939

**Published:** 2021-08-11

**Authors:** Susanna Esposito, Bahaa Abu-Raya, Paolo Bonanni, Fabianne Cahn-Sellem, Katie L. Flanagan, Federico Martinon Torres, Asuncion Mejias, Simon Nadel, Marco A. P. Safadi, Arne Simon

**Affiliations:** ^1^Pediatric Clinic, University Hospital, Department of Medicine and Surgery, University of Parma, Parma, Italy; ^2^Department of Pediatrics, University of British Columbia, Vancouver, BC, Canada; ^3^Specialization Medical School of Hygiene, Department of Health Sciences, University of Florence, Florence, Italy; ^4^Association Française de Pédiatrie Ambulatoire (AFPA), Paris, France; ^5^Tasmanian Vaccine Trial Centre, Launceston General Hospital, Launceston, TAS, Australia; ^6^School of Medicine, University of Tasmania, Launceston, TAS, Australia; ^7^Department of Immunology and Pathology, Monash University, Melbourne, VIC, Australia; ^8^School of Health and Biomedical Science, Royal Melbourne Institute of Technology (RMIT) University, Melbourne, VIC, Australia; ^9^Translational Pediatrics and Infectious Diseases, Pediatrics Department, Hospital Clínico Universitario de Santiago de Compostela, Santiago de Compostela, Spain; ^10^Genetics, Vaccines and Pediatrics Research Group, Instituto de Investigación Sanitaria de Santiago de Compostela, Universidad de Santiago, Santiago de Compostela, Spain; ^11^Division of Infectious Diseases, Department of Pediatrics, Center for Vaccines and Immunity Nationwide Children’s Hospital-The Ohio State University College of Medicine, Columbus, OH, United States; ^12^Department of Pharmacology and Pediatrics, Malaga Medical School, Malaga University, Malaga, Spain; ^13^St. Mary’s Hospital, London, United Kingdom; ^14^Department of Pediatrics, Santa Casa de Sao Paulo School of Medical Sciences, Sao Paulo, Brazil; ^15^Klinik für Pädiatrische Onkologie und Hämatologie Universitätsklinikum des Saarlandes, Homburg, Germany

**Keywords:** monoclonal antibodies, nirsevimab, palivizumab, vaccine, RSV

## Abstract

Routine childhood vaccinations are key for the protection of children from a variety of serious and potentially fatal diseases. Current pediatric vaccine schedules mainly cover active vaccines. Active vaccination in infants is a highly effective approach against several infectious diseases; however, thus far, for some important viral pathogens, including respiratory syncytial virus (RSV), vaccine development and license by healthcare authorities have not been accomplished. Nirsevimab is a human-derived, highly potent monoclonal antibody (mAb) with an extended half-life for RSV prophylaxis in all infants. In this manuscript, we consider the potential implications for the introduction of an anti-viral mAb, such as nirsevimab, into the routine pediatric vaccine schedule, as well as considerations for coadministration. Specifically, we present evidence on the general mechanism of action of anti-viral mAbs and experience with palivizumab, the only approved mAb for the prevention of RSV infection in preterm infants, infants with chronic lung disease of prematurity and certain infants with hemodynamically significant heart disease. Palivizumab has been used for over two decades in infants who also receive routine vaccinations without any alerts concerning the safety and efficacy of coadministration. Immunization guidelines (Advisory Committee on Immunization Practices, Joint Committee on Vaccination and Immunization, National Advisory Committee on Immunization, Centers for Disease Control and Prevention, American Academy of Pediatrics, The Association of the Scientific Medical Societies in Germany) support coadministration of palivizumab with routine pediatric vaccines, noting that immunobiologics, such as palivizumab, do not interfere with the immune response to licensed live or inactivated active vaccines. Based on the mechanism of action of the new generation of anti-viral mAbs, such as nirsevimab, which is highly specific targeting viral antigenic sites, it is unlikely that it could interfere with the immune response to other vaccines. Taken together, we anticipate that nirsevimab could be concomitantly administered to infants with routine pediatric vaccines during the same clinic visit.

## Background

Vaccination is a highly effective strategy to reduce the morbidity and mortality of many infectious diseases. Currently, vaccination prevents 2–3 million deaths yearly from diseases such as diphtheria, tetanus, influenza, Hepatitis B, *Haemophilus influenzae* b (Hib), pertussis, Yellow fever, poliomyelitis and measles, and saves almost 97 million disability-adjusted life years (1, 2). Highlighting the importance of vaccination as a public health intervention, the Advisory Committee on Immunization Practices (ACIP) of the US Centres for Disease Control and Prevention recommends routine immunization against 17 vaccine-preventable diseases in infants, children, adolescents or adults (3). Children are particularly vulnerable to infections, and pediatric vaccines have dramatically reduced childhood mortality due to infectious diseases (4, 5). Since 1990, mortality in children 5–9 years of age has decreased by 61% due to a reduction in the incidence of infectious diseases (6). Over the last decade, more than 1 billion children have been vaccinated against infectious diseases and during 2019, around 85% of infants worldwide received three doses of the diphtheria-tetanus-pertussis (DTP) vaccine (2).

Although active infant vaccination is highly effective for a number of viral diseases (e.g., rotavirus, polio, measles, mumps, rubella and chickenpox), the development of effective vaccines against certain viral pathogens (e.g. human immunodeficiency virus [HIV], respiratory syncytial virus (RSV), hepatitis C, human cytomegalovirus) has not been successful so far (7). Passive immunization approaches with monoclonal antibodies (mAbs) could be considered for introduction into routine pediatric vaccination schedules (7), that currently include live and inactivated active vaccines. Several mAb-related clinical trials are being conducted in different countries around the world. However, only for few of them, data adequate to allow authorization by Food and Drug Administration and European Medicines Agency have been collected with well performed clinical trial. Most are in a very early stage and cannot be adequately evaluated (8). Although mAbs are one of the fastest-growing drug classes in last years, their precise mechanism of action is yet unknown. Any outcome with therapeutic mAb is related to several factors. Important factors include antigen cell-surface density, tissue distribution, specificity, avidity, and isotype (9). The reason for the slow speed in developing mAbs include unreasonable costing for research and development, especially when compared with small molecule drugs and vaccines (10). Additionally, the complexity and ambiguity of viruses as associated with their rapid mutation make it difficult for researchers to develop effective and long-lasting mAb therapy (11).

Typically, the introduction of a new active vaccine requires data on co-administration with passive vaccines with which it will be given, to ensure non-interference with immunity (12). There is no specific guidance regarding the introduction of mAbs for use with routine pediatric vaccines. To address this topic, consideration is given to the mechanism of action of antiviral mAbs and cumulated experience with palivizumab, the only marketed mAb used for prevention of serious lower respiratory tract disease caused by RSV in the pediatric population.

## Respiratory Syncytial Virus: Disease Burden

RSV is the most common cause of acute lower respiratory infection (i.e. pneumonia and bronchiolitis) in infants and young children with most children experiencing at least one episode of RSV infection in the first 2 years of life (13). Consequently, RSV is a leading cause for infant hospitalization worldwide and is also responsible for a large number of outpatient and primary care visits contributing to substantial economic burden (14–17). In low- and middle-income countries, RSV is also a primary cause of infant mortality (18). It was estimated that globally in 2015, RSV lower respiratory tract infections were responsible for as many as 118200 deaths in children < 5 years of age (13). Apart from the acute morbidity, RSV can lead to long-term consequences; as it has been associated with subsequent development of wheezing or asthma in childhood (19–21), although it is still unclear if this association is causal (22). In line with this, there is a significant impact in the long-term healthcare resource utilization that last years following an RSV infection that occurred in the first year of life.

The burden imposed in the healthcare system post-RSV infection extends across all gestational ages and leads to a high economic burden through hospitalizations, physicians and emergency department visits (23). The majority of hospitalized infants due to RSV (67%–79%) are otherwise healthy with no underlying conditions, highlighting that severe RSV infection can occur in all infants (24–26). There is no available vaccines or definite antiviral treatment for RSV infection and clinical management is focused on symptomatic relief and supportive care (27). The only prophylactic agent currently approved against RSV is palivizumab, which is indicated only in specific groups of preterm infants or infants with comorbidities [i.e., chronic lung disease (CLD), hemodynamically significant congenital heart disease] (28). Therefore, there is a high unmet medical need for the prevention and management of RSV disease in all infants.

## Passive Immunization

Passive immunization with mAbs is promising strategy for protecting infants from infections. mAbs can be manufactured *in vitro* in large quantities with high specificity and consistency, and minimal biohazard potential (29, 30). In recent years, the development of highly potent human mAbs provide new opportunities for prophylaxis. These antibodies have primarily been isolated from suitable donors *via* large-scale screening and single B cell-based methods (7, 31). Furthermore, the antibodies may be engineered to increase their neutralization potency, extend their half-life, and alter their effector function, to provide a fine-tuned antibody designed to neutralize a specific pathogen both effectively and safely. In particular, the combination of increased potency and extended half-life mAbs affects dosing; enhanced potency means that less antibody is required to neutralize the antigen and reach the effective concentration, and extended half-life maintains that effective concentration for a longer duration. This may result in a lower dose (in terms of antibody concentration and fluid volume), allowing for easier administration (i.e., IM injection rather than IV infusion) (32), and less frequent dosing (half-life extension may require antibody administration only every ~3–6 months to maintain antibody effectiveness) (7).

Immunization in pregnancy is an alternative strategy to achieve passive immunization in infants and has proven to protect some vaccine-preventable diseases in infancy (33–35). Protection to the infant is provided *via* the active transplacental transfer of antigen-specific maternal IgG (34), which leads to passive immunization for the first months of the infant’s life (36). Immunization in pregnancy has proven to be an effective strategy for protecting infants against influenza, tetanus and pertussis (34), in the first few months of life when infants are susceptible to infections, severe disease and death (37). Hence, inactivated influenza and tetanus toxoid and acellular pertussis vaccines, which offer protection in infancy (34), are recommended during pregnancy in the United Kingdom, the United States and other countries (38, 39).

Although there is increasing evidence to support vaccination in pregnancy due to its demonstrated benefits on prevention of influenza and pertussis in infants in the first 6 months of life (40), maternal immunization offers transient protection (36), that can be variable as it depends on the levels of antibodies transferred to the infant, which in turn depends on the levels of antibodies produced by the mother (34, 40). Maternal immunization could interfere with vaccine responses in infants. A recent study showed that maternal immunization with tetanus-diphtheria-acellular-pertussis was associated with reduced antibody responses to diphtheria and pertussis, and also pneumococcus vaccine antigens that were administered as routine immunizations in the first year of life, although the clinical significance of these findings are uncertain (41). For diseases with a seasonal pattern (e.g. RSV), appropriate timing of immune protection is key and needs to be adapted to the seasonality of the virus (7). Importantly, a recent large randomized controlled clinical trial, in which pregnant women received an RSV nanoparticle-based vaccine, failed to meet the pre-specified criterion of reduction of medically significant RSV infection in infants during the first 90 days of life, although the suggestion of a possible benefit with respect to other end-point events involving RSV-associated respiratory disease in infants (42–44).

### Palivizumab

The only approved mAb for prophylactic protection against RSV in preterm infants and infants with comorbidities is palivizumab (Astra Zeneca/Medimmune). Palivizumab is a humanized mAb, which targets antigenic site II located in the pre and post fusion (F) conformations of the RSV F protein and has a half-life of 19–27 days (45). It requires monthly injections to maintain protection during the typical 5-month RSV season, at a dose of 15 mg/kg of body weight (28, 46, 47). It has shown efficacy against RSV disease by reducing the risk of hospitalization by 39–78% in groups of infants who are susceptible to severe RSV disease (48, 49). In the European Union (EU) and US, palivizumab was approved for use in preterm infants (≤35 weeks gestational age) who are aged <6 months, children with chronic lung disease (CLD) <2 years, and children with hemodynamically significant congenital heart disease (CHD) aged <2 years (46, 47). The American Academy of Pediatrics (AAP) has subsequently restricted the use of palivizumab to preterm infants with a gestational age of < 29 weeks (50), infants < 32 weeks with CLD, and those with CHD, limiting RSV prophylaxis to <2% of the annual US birth cohort (51). Recommendations for prophylaxis with palivizumab in preterm infants without comorbidities, which take into account the role of environmental risk factors, exist in several European countries (e.g. Italy, Spain, Germany) (52–54).

### Nirsevimab

Nirsevimab is a mAb that has been developed for the prevention of RSV disease, which is currently in Phase 3 trials. It is a highly potent, recombinant human mAb that specifically targets site Ø in the prefusion conformation of the F protein. Nirsevimab was functionally optimized from its parental antibody D25, originally derived from a human B cell (55, 56). It neutralizes a diverse panel of both RSV A and B strains *in vitro* with a >50-fold higher potency than palivizumab. Furthermore, it contains a three amino acid YTE substitution in the Fc region, resulting in an extended half-life in comparison with palivizumab (45), which could translate into durable protection throughout 150 days instead of 19-27 days (55).

In a Phase 1b/2a dose-escalation study, target serum concentrations remained above the 90% effective concentration level of 6.8 μg/mL in 87% of infants who received a 50 mg dose of nirsevimab and 90% of healthy preterm infants receiving this dose showed a ≥4-fold rise from baseline in serum RSV-neutralizing antibody levels. In addition, nirsevimab was well tolerated at all study doses tested (55). In a randomized, placebo-controlled Phase 2b study, nirsevimab reduced the incidence of medically attended RSV-associated lower respiratory tract infections (LRTI) by 70.1% [95% confidence intervals (CI): 52.3, 81.2], as well as the incidence of hospitalization for RSV-associated LRTI by 78.4% (95% CI: 51.9, 90.3) compared with placebo in healthy preterm infants (2.6% *vs* 9.5%, p<0.0001, and 0.8% *vs* 4.1%, p<0.0001, respectively). These differences were consistently observed throughout the 150-day period following nirsevimab administration across different geographical locations and for both RSV A and B subtypes (57). The incidence of non-RSV LRTI was similar between the nirsevimab- and placebo arms, suggesting that nirsevimab did not modify the prevalence of infections caused by other respiratory pathogens. Furthermore, the incidence of antidrug antibodies (ADA) against RSV following administration of nirsevimab was low (5.6% *vs* 3.8% for placebo) with no difference in safety results between groups when analyzed by positive or negative ADA status (57). Nirsevimab is currently in Phase 3 trials for RSV prevention in healthy full-term and late-preterm infants >35 weeks’ gestation, as well as in preterm infants ≤35 weeks’ gestation who are eligible for palivizumab administration (Phase 2/3) (58, 59).

Prophylaxis with RSV-specific mAbs has shown to be protective in specific risk groups with palivizumab, and the results from the nirsevimab study support its continued development for the wider prevention of RSV infection in healthy infants. If proven safe and efficacious also in healthy newborns, nirsevimab might be incorporated into the routine immunization program. Nirsevimab implementation might be based on a different strategy for infants born in the RSV season compared with those born out of season. For infants born during the RSV season, nirsevimab could be given as soon as possible after birth in advance of most routine vaccines. For infants born outside the RSV season, nirsevimab might need to be administered concomitantly with other recommended pediatric vaccines during routine vaccination visits, as this approach would minimize clinic visits and increase compliance (60).

## Vaccination Schedule: Examples From the US and Europe

Optimal response to a vaccine depends on various factors, including vaccine type, age and immune status of the host. Recommendation on vaccine administration depends on age-specific risks for the development of the disease, complications and responses to vaccination, as well as potential interference with the immune response by passively-transferred maternal antibodies. In general, vaccination is recommended for the age group which is the youngest at risk of experiencing the disease for which the vaccine has shown efficacy and safety (3).

In the US, the vaccination schedule in children and adolescents (<18 years of age) includes administration of vaccines against hepatitis B; rotavirus; diphtheria, tetanus and pertussis; Hib; pneumococcal disease; poliovirus; influenza; hepatitis A; measles, mumps and rubella (MMR); varicella; HPV and meningococcal disease as recommended by the CDC (**Table 1**). A catch-up immunization schedule for children aged 4 months to 18 years who start late or are >1 month behind exists providing minimal intervals between doses for children based on age who have experienced delay in vaccination. Specific recommendations exist for certain high-risk groups (e.g. children undergoing chemotherapy or radiation treatment, children with HIV) (61). In Latin America, beyond all these vaccines, immunization programmes in most countries also include BCG vaccine in the neonatal period as well as yellow fever vaccine in countries where the disease is endemic (62). In Europe, the vaccination schedules between different countries are similar, but there are differences in terms of the dosing, the exact type of vaccine and whether a vaccine is administered alone or in combination with other vaccines. Childhood vaccination schedules in all EU/European Economic Area (EEA) countries include vaccination against MMR, DTP, poliomyelitis, Hib and human papillomavirus (adolescent, pre-adolescent girls) (63).

**Table 1:** Main vaccines included in the US vaccination schedule for children aged <18 years (CDC, 2020) (56)^a^.

Vaccine	Birth	1–2 mo	4 mo	6–9 mo	12–18 mo	19–23 mo	4–6 yr	11–12 yr
**Hepatitis B**	1^st^ dose	2^nd^ dose	3^rd^ dose						
**Rotavirus**		1^st^ dose (2 mo)	2^nd^ dose	3^rd^ dose (6 mo)^b^				
**DTaP**		1^st^ dose (2 mo)	2^nd^ dose	3^rd^ dose (6 mo)	4^th^ dose (15–18 mo)		5^th^ dose	
**Hib**		1^st^ dose (2 mo)	2^nd^ dose	3^rd^ dose^c^ (6 mo)	3^rd^ or 4^th^ dose (12–15 mo)			
**Pneumococcal conjugate**		1^st^ dose (2 mo)	2^nd^ dose	3^rd^ dose (6 mo)	4^th^ dose (12–15 mo)			
**Inactivated poliovirus**		1^st^ dose (2 mo)	2^nd^ dose	3^rd^ dose			4^th^ dose	
**Influenza (IIV)**				Annual vaccination 1 or 2 doses (6 mo–18 yr)				
**MMR**					1^st^ dose (12–15 mo)		2^nd^ dose	
**Varicella**					1^st^ dose (12–15 mo)		2^nd^ dose	
**Hepatitis A**					2-dose series			
**MenACWY-D, MenACWY-CRM or MenACWY-TT**								1^st^ dose and 2^nd^ dose (16 yr)
**HPV**								2- or 3-dose series depending on age at initial vaccination

^a^Does not include recommendations for high-risk groups; ^b^For RotaTeq; ^c^For ActHIB, Hiberix, or Pentacel.

DTaP, diphtheria tetanus & acellular pertussis (< 7 yrs); Hib, Haemophilus influenzae type b; HPV, human papillomavirus; IIV, inactivated influenza virus; mo, month; MenACWY, meningococcal vaccine ACWY; MMR, measles mumps rubella; yrs, years.

Although the World Health Organization (WHO) recommends vaccination against hepatitis B (64), some EU/EEA countries offer this vaccine only in high-risk groups (63). In some EU/EEA countries, the vaccination schedule includes immunization against hepatitis A, influenza, invasive disease caused by *Neisseria meningitidis* or *Streptococcus pneumoniae*, rotavirus, tuberculosis and varicella (63).

## Coadministration of Pediatric Vaccines

Coadministration or simultaneous administration of vaccines is defined as administering more than one vaccine on the same clinic day, at different anatomic sites, not combined in the same syringe (3). Coadministration of all vaccines that a child is eligible for at the time of a clinic visit is key for ensuring timely vaccination and also for bringing children up to date (62). The value of pediatric vaccine coadministration, to avoid missing recommended vaccinations, is highlighted by a study investigating a measles outbreak among unvaccinated children aged <5 years in the US. The study found that 38% of these children had received vaccinations against DTP and pertussis and/or oral poliovirus at a time when they could also have simultaneously received measles vaccination (65).

Several studies have investigated the efficacy/immunogenicity and safety of pediatric vaccines administered simultaneously and support current recommendations (ACIP, AAP and WHO) on coadministration of vaccines in children (3, 66, 67). Studies addressing the coadministration of different vaccines, such as MMR with DTP and oral poliovirus, Hib with either MMR or DTP and oral poliovirus, and hepatitis B with DT and oral poliovirus, have demonstrated that simultaneous administration of commonly used live and inactivated vaccines results in seroconversion rates and rates of adverse events that are similar to those following single administration (3, 68, 69). Coadministration of routine pediatric vaccines is recommended for children with no contraindications at the time of vaccination (3). Examples of coadministered vaccines showing no evidence of interference with the immunologic response to any of the antigens include MMR and varicella vaccines; live attenuated influenza and MMR or varicella vaccines; PPSV and inactivated influenza vaccines (IIV); hepatitis B and yellow fever vaccines (3, 70). Although no data are available on the immunogenicity of the Ty21a typhoid vaccine when administered simultaneously or within 30 days of live-virus vaccination, if typhoid vaccination is recommended, it should not be delayed due to recent receipt of live-virus vaccines (3). Furthermore, the ACIP still recommends that IIV and PCV13 are administered simultaneously following a benefit–risk assessment based on the 2010–2011 influenza season data (3, 71, 72).

There are few exceptions to the recommendation of coadministration of pediatric vaccines. In patients with anatomic or functional asplenia and/or HIV, the quadrivalent meningococcal conjugate vaccine MCV4-D (MenACWY-D) and the pneumococcal conjugate vaccine PCV13 should not be administered concomitantly (3). This contraindication is based on data from immunocompetent children showing reduced antibody concentrations (anti-pneumococcal IgG) for vaccine serotypes following coadministration of MenACWY-D and PCV7 compared with PCV7 alone (3). Therefore, due to their baseline high risk of invasive pneumococcal disease, children with anatomic or functional asplenia and/or HIV should not receive vaccination against MenACWY-D concomitantly with PCV-13 before 2 years of age. If these children are older than 2 years and have not yet received all recommended doses of PCV, they should receive all the doses separately from MenACWY-D by at least 4 weeks (3, 73, 74).

Although current experience supports coadministration of vaccines, the introduction of a new vaccine is typically supported by data demonstrating that the vaccine does not interfere with the immune response to other vaccines that are likely to be administered at the same visit (75). While the European Medicines Agency (EMA) does not generally require vaccine coadministration studies to be conducted prior to licensure (75), vaccine use may be limited until data are available on coadministration with types of vaccines to be given at the same time (76). The Food and Drug Administration (FDA) recommends including an assessment of coadministration with relevant vaccines in Phase 3 trials to support vaccine licensure and appropriate label claim (12).

## Coadministration of Anti-Viral Monoclonal Antibodies With Pediatric Vaccines

There is limited published evidence on the coadministration of mAbs with pediatric vaccines. The mechanism of action of mAbs might provide insights into the potential for an anti-viral mAb to interfere with the immune response to a vaccine. As described above, most mAbs are human antibodies which exhibit high potency and expected specificity against viral pathogens and are often engineered to further enhance their *in vivo* functions (7, 31). Anti-viral effects of antibodies can be mediated *via* several mechanisms. The mechanism that targets free viral particles and is associated with protection *in vivo* is neutralization, which is measured *in vitro* as the ability of an antibody to prevent viral entry into target cells. Effector functions mostly mediated *via* the Fc portion of antibodies include antibody-dependent cellular cytotoxicity (ADCC), complement-dependent cytotoxicity, antibody-dependent cellular phagocytosis (ADCP), antibody-dependent neutrophil phagocytosis and it is believed that these mechanisms may also play a key role in mediating protection against the pathogen. As neutralization often correlates with the ability to bind to native structures on the viral surface, it can give some indication of the ability of antibodies to mediate effector activities such as ADCC and ADCP (7).

Based on the mechanism of action of nirsevimab, which specifically neutralizes RSV preF to provide protection *in vivo*, its effects are not expected to interfere with the immune response to other vaccines (77). However, mAb–vaccine interaction studies are generally not required by regulatory authorities to support licensure indicating the lack of necessity or clinical relevance of such evaluations. This is reflected in regulatory guidelines. Neither the FDA nor EMA guidelines on clinical evaluation of products for RSV prophylaxis recommend trials to assess coadministration with other vaccines for mAbs. The recent guidance issued by the EMA and FDA covering the clinical development of RSV mAbs for prophylaxis neither require or suggest conducting coadministration studies with other vaccines, although the EMA guidance does include such recommendations for development of RSV vaccines (75, 76). FDA guidance on assessing potential drug interactions for therapeutic proteins advises to consider the potential mechanism for drug-drug interactions taking into consideration the pharmacology of the drug and potential coadministered drugs in the patient population (78). For an anti-viral mAb there is no yet potential mechanism for interference with the immune response to vaccines targeting other viruses or bacteria (e.g. bezlotoxumab) (79). To date, the conduct of mAb–vaccine interference studies has been limited to mAbs directed against immune mediators to assess if the mAb would alter the function of the immune system and affect the response to vaccinations in the patient population (e.g. secukinumab, dupilumab) (46, 80, 81). However, there aren’t large enough studies to determine if mAbs influence immune functions. WHO recommendations (draft version) regarding the preferred product characteristics of mAbs for passive immunization against RSV, state that there is *“no significant negative impact on immune responses to co-delivered vaccines”* (82).

There are limited country-specific guidelines focusing on the coadministration of antibodies and vaccines. Polyclonal intravenous Ig (IVIG) might interfere with the immune response to many live vaccine viruses, as antibodies may prevent replication of the vaccine virus. Therefore, live viral vaccines should be administered at least three weeks before or 3 to 11 months after an injection of polyclonal IVIG depending on the dose, with exceptions to these recommendations being vaccines against yellow fever, rubella and BCG (83). However, these recommendations are not based on the use of highly specific mAbs. In France, coadministration of pediatric vaccines is possible for DTP, Hib, hepatitis B and pneumococcus at 2 months and 4 months of age, meningococcus C and MMR at 12 months of age, as well as Tdap and HPV at 11–13 years of age; however, further details on coadministration of vaccines or vaccines and antibodies are not provided (84). In general, the administration of live viral vaccines within 3–6 months of polyclonal IVIG is discouraged, as the IVIG product may contain IgG antibodies, that could neutralize the live virus in the vaccine and impair vaccine uptake (85). However, this is less relevant in the case of mAbs specific for a particular pathogen, unless that same specific pathogen is included in the live virus vaccine (86, 87).

There is limited published evidence on the coadministration of palivizumab with pediatric vaccines; only one observational study from Japan has investigated the effects of coadministration in infants and confirmed that there are no safety concerns (88). Guideline recommendations on palivizumab use are consistent and support its coadministration with pediatric vaccines. The ACIP states the following: *“A humanized mouse monoclonal antibody product (palivizumab) is available as prophylaxis for serious lower respiratory tract disease from RSV among infants and young children. This product contains only antibody to RSV and does not interfere with the immune response to licensed live or inactivated vaccines”* (3). Recommendations by the Joint Committee on Vaccination and Immunization (JCVI) in the UK state that palivizumab “*can be given at the same time as vaccines administered as part of the routine childhood immunization programme”* (83). According to the National Advisory Committee on Immunization (NACI) in Canada: *“Palivizumab is a passive immunizing agent with a highly specific antigen target (the F glycoprotein of RSV). It therefore does not interfere with the immune response to vaccines and can be administered at the same time at a separate site”* (88). Similarly, based on the CDC Pink Book: “*While certain antibody products like immune globulins interfere with live-virus vaccines, monoclonal antibody products specific to one, non-vaccine microbe do not interfere with live vaccines. Since palivizumab does not contain any other antibody except RSV antibody, it will not interfere with the response to a live virus vaccine”* (89). In addition, according to the AAP: *“Palivizumab does not interfere with the immune response to live or inactivated vaccines. The childhood immunization schedule should be followed for all children, regardless of palivizumab use”* (90). The Association of the Scientific Medical Societies (AWMF) in Germany states that “*Prophylaxis with palivizumab can be given at the same time as active vaccinations”* (54).

No formal drug–drug or drug–vaccine interaction studies were conducted for palivizumab (47). During clinical development, palivizumab safety was assessed by vaccination status. Based on the very specific mechanism of action of RSV mAbs, safety assessments by vaccination status in clinical development are adequate to support licensure and co-administration with pediatric vaccines. In line with guideline recommendations, it should be noted that in clinical trials using palivizumab, the proportion of children in the placebo and palivizumab groups who received routine childhood vaccines or influenza vaccine were similar, with no increase in adverse reactions between the groups (47, 49). Furthermore, palivizumab has been used for over 22 years in preterm infants and infants with CHD or CLD who also receive routine pediatric vaccinations (FDA and EMA approval in 1998 and 1999, respectively).

There have been limited safety concerns due to palivizumab concomitant administration with other vaccines based on real-world evidence. Notably, the Phase 2b trial of nirsevimab allowed routine pediatric vaccinations to take place as planned and therefore, the efficacy and safety of nirsevimab looking at RSV and non-RSV infections was demonstrated in the context of infants receiving other pediatric vaccines (57). The ongoing nirsevimab Phase 3 trial will also allow administration of routine pediatric vaccines (58, 59). However, some attention should be given to immune enhancement, as exemplified by flavivisus (91). Palivizumab in infancy in otherwise healthy preterm infants has been associated with persistent effects on the abundance of specific, potentially pathogenic, microbial taxa in the respiratory tract, although the meaning of this effect is unclear (92–94).

## Conclusions

While specific studies investigating the coadministration of anti-viral mAbs with vaccines have not been performed, the mechanism of action of mAbs, which in the case of viruses is based on targeting highly specific viral antigenic sites, does not raise any concerns regarding coadministration with vaccines. In line with this, palivizumab, the only approved mAb for prophylactic use in preterm infants and infants with comorbidities to protect against severe RSV infection, has been used for more than two decades in infants who also receive routine vaccinations. To date, concerns related to vaccine efficacy or safety have not been reported. In addition, various guidelines (ACIP, JCVI, NACI, CDC, AAP, AWMF) support coadministration of palivizumab with routine pediatric vaccines, as it is highly unlikely that palivizumab interferes with the immune response to other vaccines. Based on the evidence discussed in this white paper and the mechanism of action of nirsevimab, it is not anticipated that coadministration of nirsevimab with routine pediatric vaccines will interfere with the immune response to the vaccine antigens. Therefore, although future studies on efficacy and safety of coadministration of nirsevimab with routine pediatric vaccines are important as well as there is the need to call for research on the administration of mAbs on vaccination and immunity, nirsevimab could be given to infants concomitantly with routine vaccinations during the same clinic visit unless evidence suggests otherwise.

## Author Contributions

SE proposed the project, coordinated the study group, and wrote the first draft of the manuscript. BA-R, PB, FC-S, KF, FM, AM, SN, MS, and AS revised the initial draft of the manuscript, provided comments, and substantially contributed to the content of the manuscript. All authors contributed to the article and approved the submitted version.

## Funding

The World Association of Infectious Diseases and Immunological Disorders received an unrestricted grant from Sanofi Pasteur for research and medical education activities on RSV monoclonal antibodies and co-administration with pediatric vaccines (Waidid_2021_03). The funder was not involved in the study design, collection, analysis, interpretation of data, the writing of this article or the decision to submit it for publication.

## Conflict of Interest

SE: Research support from GSK, Sanofi, and Vifor. Speaker’s fees from GSK, Janssen, Pfizer, Novartis, Sanofi Pasteur, and MSD in the past 3 years. BA is supported by the Canadian Health and Research Institute Vanier Canada scholarship. PB received grants for epidemiological and HTA research projects from GlaxoSmithKline, MSD, Sanofi Pasteur, Pfizer, Seqirus and Astra Zeneca, and fees for taking part in advisory boards on different vaccines from the same companies plus Janssen. FM has received honoraria from GSK, Pfizer, Sanofi Pasteur, MSD, Seqirus, and Janssen for taking part in advisory boards and expert meetings, and for acting as speaker in congresses outside the scope of the submitted work. FM has also acted as principal investigator in RCTs of the above-mentioned companies as well as Ablynx, Regeneron, Roche, Abbot, Novavax, and Medimmune, with honoraria paid to his institution. FM research activities received support from the Instituto de Salud Carlos III (Proyecto de Investigación en Salud, Acción Estratégica en Salud): project ReSVinext ISCIII/PI16/01569/Cofinanciado FEDER and project Enterogen (ISCIII/PI19/01090). KF is a member of the Australian Technical Advisory Group on Immunisation (ATAGI) but the views in this manuscript are her own and not necessarily those of ATAGI. KF has received honoraria as a member of the vaccine advisory boards for Seqiris and Sanofi Pasteur in the last 5 years. AM has received research grants from NIH, Janssen and Merck AND fees for participation in advisory boards from Janssen, Sanofi-Pasteur, Merck and Roche. MS reports research grants and personal fees for advisory boards from GSK, Pfizer, Sanofi-Pasteur, Janssen and Seqirus.

The remaining authors declare that the research was conducted in the absence of any commercial or financial relationships that could be construed as a potential conflict of interest.

## Publisher’s Note

All claims expressed in this article are solely those of the authors and do not necessarily represent those of their affiliated organizations, or those of the publisher, the editors and the reviewers. Any product that may be evaluated in this article, or claim that may be made by its manufacturer, is not guaranteed or endorsed by the publisher.
